# Detection of human strongyloidiasis among patients with a high risk of complications attending selected tertiary care hospitals in Colombo, Sri Lanka using molecular and serological diagnostic tools

**DOI:** 10.1186/s13071-024-06508-x

**Published:** 2024-10-12

**Authors:** Chamarika Jayanetti Weerasekera, Nayana Gunathilaka, Chandrani Menike, Philip Anpahalan, Nilanka Perera, Nilanthi Renuka de Silva, Renu Wickremasinghe

**Affiliations:** 1 University of Sri Jayewardenepura, Nugegoda, Sri Lanka; 2 University of Kelaniya, Ragama, Sri Lanka; 3 Ministry of Health, Colombo, Sri Lanka

**Keywords:** Immunocompromised, Molecular diagnosis, Parasitological diagnosis, Serological diagnosis, Soil-transmitted helminth, Strongyloidiasis, *Strongyloides stercoralis*

## Abstract

**Background:**

Strongyloidiasis a neglected tropical disease is known to cause severe disease among immunosuppressed and has not been studied extensively in Sri Lanka. Parasitological diagnostic approaches based on faecal microscopy and culture often fail to detect low-intensity infections. This study investigates the presence of strongyloidiasis among selected immunocompromised individuals using parasitological, molecular and serological techniques.

**Methods:**

Adult patients with immunocompromising conditions admitted to three tertiary care hospitals in Sri Lanka were recruited. A faecal sample and 2 ml of venous blood were collected. The faecal samples were subjected to direct faecal smear and cultures (agar plate, charcoal and Harada-Mori) and polymerase chain reaction (PCR) using species specific primers designed for *Strongyloides stercoralis*. The presence of *Strongyloides* IgG antibodies was tested in the collected serum samples using DRG *Strongyloides* IgG enzyme-linked immunosorbent assay (ELISA) kits. The PCR products of the positive samples were sequenced using Sanger sequencing method.

**Results:**

A total of 260 patients were recruited to this study, out of which 160 provided faecal samples and 122 provided blood samples. Out of the 160 faecal samples, none were positive for strongyloidiasis by direct smear, charcoal and Harada-Mori cultures. Only one sample (0.6%) was positive by agar plate culture. Out of the 123 samples subjected to PCR, 14 (11.4%), including the culture positive patient, were positive for *S. stercoralis*. Sequencing results of the PCR products indicated 100% similarity to *S. stercoralis*. Out of the 122 serum samples subjected to ELISA, 20 (16.4%), including the culture positive patient, were positive for *Strongyloides* IgG antibodies. However, sociodemographic, exposure factors, clinical features were not significantly associated with the presence of strongyloidiasis infection.

**Conclusions:**

Strongyloidiasis is present among the immunocompromised population in Sri Lanka, even in the absence of a significant relationship with associated factors. It is advisable to screen such patients with highly sensitive tests such as PCR for early diagnosis and treatment.

**Graphical Abstract:**

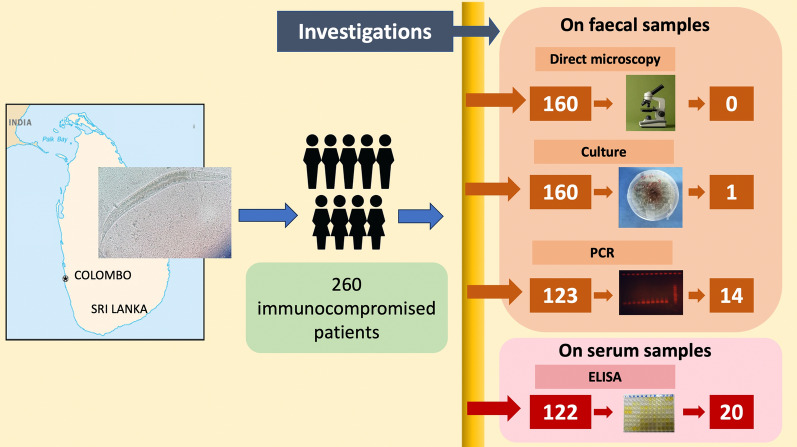

**Supplementary Information:**

The online version contains supplementary material available at 10.1186/s13071-024-06508-x.

## Background

*Strongyloides stercoralis*, a soil-transmitted helminth, is the causative organism of strongyloidiasis in humans and has been recently introduced as a neglected tropical disease (NTD) in the World Health Organization (WHO) road map for control of NTD 2021–2030 [1]. Humans acquire this infection through skin penetration by the filariform larvae of the parasite [2]. Clinical manifestations range widely from asymptomatic infection to severe disseminated disease [2]. Impairment of the host immunity, could lead to accelerated multiplication of the parasite as well as accelerated auto-infection resulting in hyper-infection with parasites within the gastrointestinal and pulmonary tract or disseminated infection, both of which could be fatal without treatment [2].

The global prevalence of strongyloidiasis is estimated to be 613.9 million (8.1%) in 2017 through statistical modelling, albeit having less sensitive diagnostic techniques that limit the sensitivity [3]. Currently, there is no accepted “gold standard” for the diagnosis of strongyloidiasis [4].

Strongyloidiasis in Sri Lanka was first reported in 1983, in a case series of two patients with watery diarrhoea [5]. In both instances, the direct smear was positive for numerous rhabditiform larvae, and the parasites were cultured through Harada-Mori technique. Since then, sporadic cases of strongyloidiasis have been reported [6–10].

Research carried out with regard to strongyloidiasis is scarce, thus there is no readily accessible data on the prevalence of strongyloidiasis in Sri Lanka [11]. A prevalence of 0.0%, 0.5% and 1.6% has been reported among children and women in the plantation sector in Sri Lanka in 1996, among children in the Jaffna municipality in 1986 and among children admitted to the paediatric unit at Jaffna Teaching Hospital in 1989, respectively [12–14]. The direct faecal smear had been the main diagnostic modality in these instances. A study conducted in 2006 has reported a prevalence rate of 11.5% among adult patients with malignancies using culture methods and 9.7% in adult patients with end stage renal disease and post renal transplant [15]. The molecular tests, by means of conventional polymerase chain reaction (PCR) was first introduced in 2020 [8].

With the general improvement of medical care as well as due to the increase of non-communicable diseases the number of immunocompromised individuals has risen both globally and locally. Transplants and administration of immunosuppressive medications (post-transplant, malignancies and connective tissue disease) occur frequently in Sri Lanka. Routine screening for parasitic disease in the immunocompromised is not being carried out in Sri Lanka currently. Strongyloidiasis is an important disease in the immunocompromised, due to the serious consequences due to hyper-infection and disseminated disease. Moreover, despite its individual shortcomings, a variety of tests are available for the diagnosis of the disease and a definitive treatment (ivermectin) is also present. Thus, it is beneficial to take this neglected STH into consideration and bridge the knowledge gaps to estimate its prevalence in the immunocompromised population and to identify associated factors and the suitable and cost-effective test methods to be carried out in resource poor settings.

Therefore, this study was conducted with the objective of identifying the presence of strongyloidiasis infection among selected immunocompromised patients in Sri Lanka using traditional coprological, molecular and serological based detection approaches and to identify the associated factors.

## Methods

### Study design and setting

This descriptive cross-sectional study was carried out over a period of 21 months (April to July 2021 and February 2022 to July 2023) by selecting patients with underlying comorbidities or treatment causing immunocompromised states at the Professorial Medical Unit, Colombo South Teaching Hospital, Kalubowila, Dehiwala, Sri Lanka, Nephrology Unit at the Sri Jayewardenepura General Hospital, Thalapathpitiya, Nugegoda, Sri Lanka, and selected wards at the Apeksha Hospital, Maharagama, Sri Lanka. The Apeksha Hospital is the National Reference Centre for patients with malignancies in Sri Lanka. Both other hospitals are major teaching hospitals closer to the city of Colombo. These hospitals were selected based on convenience, as all three hospitals are within close proximity to the laboratory, since this research was carried out during the coronavirus disease 2019 (COVID-19) pandemic and the subsequent economic and fuel crisis. Therefore, transport to far-away hospitals was very difficult. The break in the study period was due to curfews imposed due to the pandemic.

### Sample size calculation

A prevalence of approximately 10% among immunocompromised individuals was based on a study carried out in 2006 to prevent wastage of valuable resources and feasibility [15]. Therefore, the sample size was calculated using the following equation described by Lwanga and Lemeshow [16].

*N* = *Z*^2^*p* (1−*p*)/*d*^2^ [16].

*N* = sample size.

*d* = degree of accuracy (precision) desired for margin of error set at 0.05%

*Z* = standard normal deviation, set at 1.96 corresponding to 95% confidence interval.

*p* = expected proportion of disease who are immunodeficient set at 0.1

The estimated sample size was calculated to be 138. Expecting a 50% non-responder rate for the faecal samples, it was agreed to screen 280 patients for eligibility.

### Recruitment of study participants and sample collection

Patients on immunosuppressive chemotherapy for oncological or any other systemic illness or post-transplantation, on systemic radiotherapy or patients with end stage renal disease were included in the study. Those who had anthelminthic treatment less than 1 month prior to collection of samples or those with gastrointestinal bleeding or critically ill were excluded. The consented patients were requested to provide a faecal sample and a blood sample. The patients were provided with a clean, wide mouthed, screw capped container, with a small, clean, plastic spoon attached to its lid to collect the faecal samples. A venous blood sample (2 ml) was obtained from each patient to a plain tube. The collected samples were transported at room temperature to the research laboratory as the Department of Parasitology, Faculty of Medical Sciences, University of Sri Jayewardenepura. The separated serum samples and a portion of faecal samples were stored in a −80 °C freezer. The remainder of the faecal samples were processed immediately.

### Socio-demographic and risk factors for strongyloidiasis

Clinico-demographic data of the patients meeting inclusion criteria was collected using interviewer administered case report forms after obtaining informed written consent. Data on basic demographic details (age, sex and place of residence), exposure to soil such as occupation, gardening or recreational activities, basic social history for the availability of water sealed latrines, immunosuppressing condition, and presence or absence of diarrhoea were collected.

### Parasitological screening for strongyloidiasis

#### Direct microscopy of stool samples

The faecal samples were subjected to wet mount examination by saline and iodine smear by two experienced microscopists – a medical doctor specialising in Medical Parasitology and a senior medical laboratory scientist. The reference slides in the laboratory and previously harvested larvae preserved in formalin were used for comparison. Larval stage of *S. stercoralis* was examined under a compound light microscope (10x and 40×).

#### Agar plate culture

About 2–3 g of faeces were placed in a Petri dish with nutrient agar, sealed and incubated at room temperature for at least 3 days [17, 18]. At the end of incubation, the presence of tracks was observed under the dissection microscope, then the Petri dish was flooded with 5% formalin for 30 min, the liquid was pipetted out and centrifuged at 1500 rpm for 2 min and the sediment was examined under a compound light microscope.

#### Harada-Mori culture

The stool sample was emulsified and spread in the middle of a 12 cm strip of blotting paper that was as wide as a 15 ml centrifuge tube. The strip was inserted into a 15 ml centrifuge tube filled with 3–4 ml of water, sealed and incubated for 10 days at room temperature. At the end of incubation, the strip was carefully removed and discarded, and an equal volume of 10% formalin was added to the tube. The tube was centrifuged at 1500 rpm for 2 min and the sediment was examined under a compound light microscope for larval stages [17].

#### Charcoal culture

Approximately 10–20 g of faeces was mixed with an equal amount of granulated charcoal in a Petri dish and distilled water was added till charcoal glistened. The dish was incubated at room temperature for 10 days. At the end of incubation, excess water was pipetted out into a 15 ml centrifuge tube, formalin was added and centrifuged, and the sediment was examined under a compound light microscope for larval stages [19].

### Molecular based detection by polymerase chain reaction

The DNA extraction from the stool samples were carried out using the commercial QIAamp DNA Stool Mini Kit (QIAGEN, Hilden, Germany cat. no. 51504) according to the manufacturer’s instructions with some modifications (incubation with ASL buffer for 20 min, incubation with AL buffer and proteinase K for 2 h and reducing the elution volume to 100 µl). The DNA was amplified using species-specific primers targeting the *ITS1* and *5.8S* ribosomal RNA regions (**F (5' to 3'):** ATCGTGTCGGTGGATCATTCGGTT; **R (5' to 3'):** AATAGTATAAAATACTATTAGCGCCATTTGCATTC) of *S. stercoralis* with an expected band size of 129 bp [8].

The total volume per reaction was set for 20 µl, containing 10 µl 2 × HotStarTaq Plus Master Mix (QIAGEN, Hilden, Germany; cat. no. 203643), 0.12 µl each of forward and reverse primer, 2 µl of 10 × coral load concentrate, 2.76 µl of PCR water and 5 µl of template. The programme was set at 95 °C initial denaturation for 5 min followed by 40 cycles of 94 °C for 30 s (denaturation), 60 °C for 30 s (annealing), 72 °C for 28 s (extension) and final extension at 72 °C for 5 min. Negative controls with nuclease free water were run separately at each run. The electrophoresis was performed on 2% agarose gel (100 V, 200 mA for 25–30 min). A 7 µl of the products was loaded into each well. The gel was post-stained in ethidium bromide (0.5 µg/mL/TAE) for 30 min. Visualisation was done using an ultraviolet (UV) transilluminator (Maestrogen, Taiwan, and ImageJ software package NIH, USA).

### DNA sequencing and phylogenetic analysis of *S. stercoralis* isolates

The PCR products of the *S. stercoralis* positive samples were sent to Macrogen (Macrogen Inc., Seoul, Republic of Korea) for gene sequencing with same primers used in the PCR by Sanger sequencing method. The homologous sequences were explored in the GenBank database using the Basic Local Alignment Search Tool (BLAST) (National Centre for Biotechnology). The chromograms were analysed and the sequences were aligned using BioEdit 7.2 software. The evolutionary history was inferred using the neighbour-joining method. The evolutionary analyses were conducted in MEGA X1.

### Serological detection using a commercial ELISA kit for specific IgG

The presence of *Strongyloides* specific IgG antibodies were tested by means of a commercial enzyme-linked immunosorbent assay (ELISA) kit (DRG International – EIA 4208) according to the manufacturer’s instructions. The reactions were read visually as well as with the ELISA reader set at 450 nm wavelength. The test was considered valid if the optical density (OD) value for the negative control was <0.2  and ⋝0.5 for the positive control. An OD value ⋝0.2 was taken as a positive result.

### Data analysis

The data was entered in Microsoft Excel and the statistical analysis was done using R Version 4.4.1. The demographic data of the study population were analysed to assess the risk of acquisition of strongyloidiasis. Pearson chi-squared test and the Fisher’s exact test were used to assess the differences between the groups of categorical variables.

### Ethical aspects

Ethical clearance for this study was obtained from the Ethics Review Committees of Colombo South Teaching Hospital, Sri Jayewardenepura General Hospital and the Faculty of Medical Sciences of the University of Sri Jayewardenepura. Permission was sought from the relevant Directors and the relevant Consultants in charge of the unit prior to initiation of the study. Informed consent from the patients were sought to recruit them to the study.

## Results

### Socio-demographic characterization and associated factors among selected patients

**Fig. 1:**
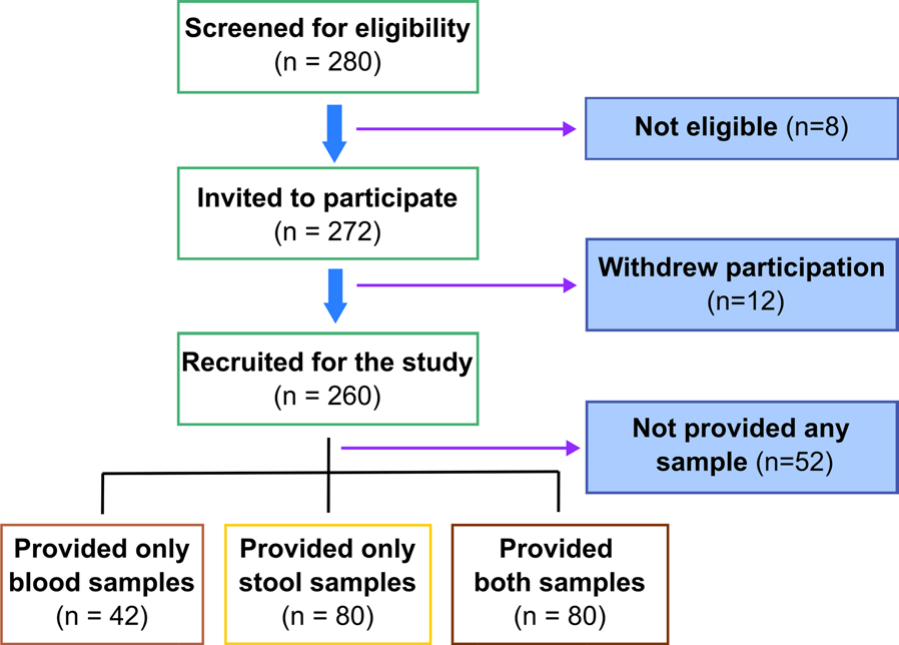
Flowchart depicting the number of patients screened, enrolled and ultimately provided samples

**Fig. 2:**
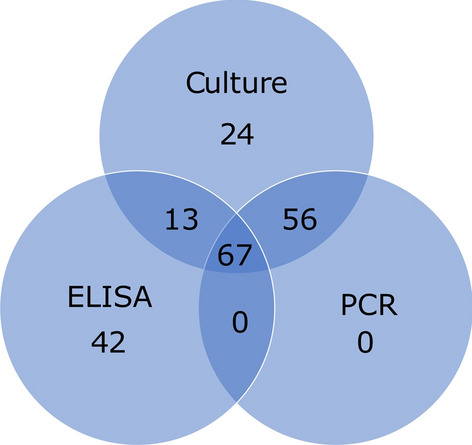
Venn diagram depicting the tests provided on the samples provided

Out of 280 patients checked for eligibility, 272 met the inclusion criteria, and out of these 260 patients consented and were recruited to the study. A total of 202 patients provided either blood or faecal sample (Fig. 1). Only 122 patients complied with blood samples, which were subjected to ELISA. One hundred and sixty (160) patients provided faecal samples, which were subjected to the direct smears and cultures. Out of the faecal samples, only 123 samples were selected randomly for conventional PCR (Fig. 2). The majority of the study participants were males (*n* = 105; 52%). The age of the patients ranged from 18 to 83 years with a mean age of 48.50 years, a median of 50 years and an inter-quartile range of 23 years. Almost 80% of the population had an education up to secondary level or above. Only patients who underwent ELISA and/or PCR were included in the final analysis. Out of the analysed study population of 178 patients, 25 had occupational soil exposure, whilst 92 had recreational exposure to soil. However, only four patients admitted to not having water-sealed latrine facility. With regards to the underlying immunosuppressing conditions, 110 had haematological malignancies followed by 31 with solid organ malignancies, 21 with end stage renal disease, eight on immunosuppressive therapy following renal transplant and eight others with various immunosuppressive conditions. In terms of clinical manifestations, 11 patients had eosinophilia and 15 patients had diarrhoea (Table 1). Overall, there was no significant association with the presence of infection and socio-demographic factors (age, sex, level of education) or exposure factors (occupational/recreational exposure to soil, barefoot walking and availability of water sealed latrines). Further, there were no significant associations observed with symptoms such as diarrhoea or presence of eosinophilia.

**Table 1:** Socio-demographic characters, associated risk factors for strongyloidiasis and clinical features of study participants

Risk factor	Positive (*n* = 33)	Negative (*n* = 145)	Statistical analysis
Gender			
Male	15	76	*X*^*2*^ = 0.28, *df* = 1,
Female	18	69	*P1* = 0.597
Age			
< 50	18	71	*χ*^2^ = 0.15, *df* = 1
> 50	15	74	*P1* = 0.698
Level of education			
Primary and below	02	14	*P2* = 0.74, OR = 0.6,
Secondary and above	21	131	95% CI 0.1–2.9
Occupational soil exposure			
No	26	127	*P2* = 0.264, OR = 0.5,
Yes	07	18	95% CI 0.19–1.65
Recreational soil exposure			
No	14	72	*χ*^2^ = 0.31, *df* = 1,
Yes	19	73	*P1* = 0.577
Foot wear			
No	05	13	*P2* = 0.335, OR = 1.8,
Yes	28	132	95% CI 0.47–1.97
Water-sealed latrines			
No	02	02	*P2* = 0.157, OR = 4.6
Yes	31	143	95% CI 0.32–65.18
Underlying immunosuppressing condition			
Malignancy (haematological and solid organ malignancy)	25	116	*χ*^2^ = 0.093, *df* = 1,
No malignancy (renal disease, post renal transplant and other)	08	29	*P1* = 0.761
Eosinophilia			
No	30	137	*P2* = 0.43, OR = 0.59
Yes	03	08	95% CI 0.13–3.63
Presence of diarrhoea			
No	32	131	*P2* = 0.31, OR = 3.4
Yes	01	14	95% CI 0.48–148.97

P1 = results by chi-squared test; P2 = results by Fisher’s exact test; *P* ≤ 0.05 statistically significant

### Parasitological screening

Both saline and iodine wet smears were checked in 160 samples and none of the direct smears (saline/ iodine) were positive for strongyloidiasis. Only one sample (0.6%) of the agar plate culture method was positive for *S. stercoralis* larvae (Additional file 1: Fig. S1), in which rhabditiform larvae were observed. However, filariform larvae or adult stages were not seen. Both Harada-Mori and charcoal cultures also indicated negative results.

### Molecular based detection

Of the 160 faecal samples, 123 samples were randomly selected for DNA extraction and molecular detection due to resource constraints. The gel electrophoresis results of the PCR product indicated 14 patients being positive for *S. stercoralis* DNA. (Additional file 2: Fig. S2).

### DNA sequencing and phylogenetic analysis of *S. stercoralis*

**Fig. 3:**
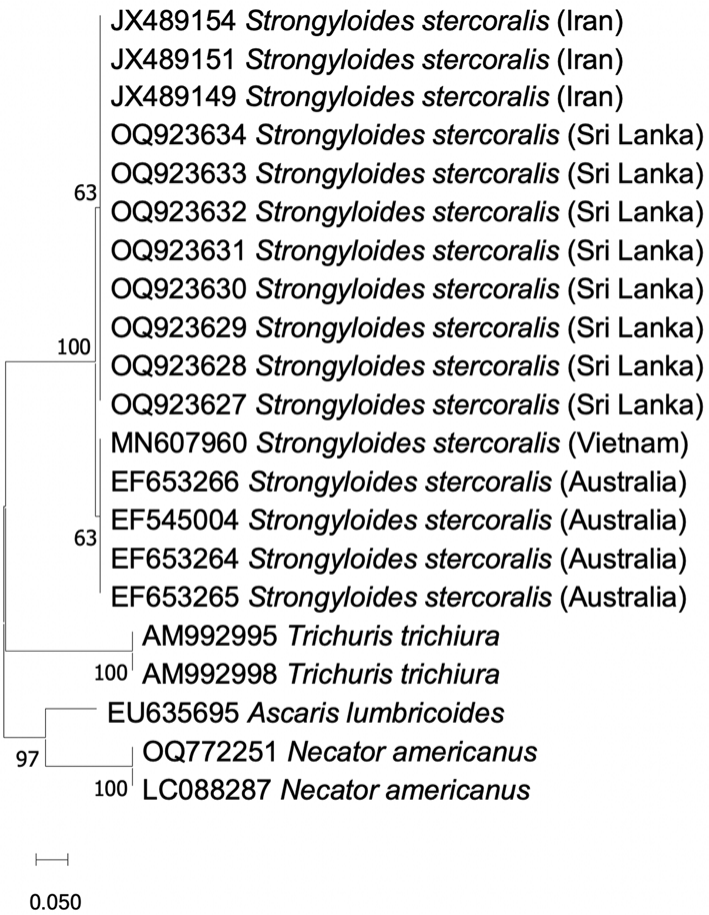
Phylogenetic relationship of new sequences isolated from the study

The new eight sequences identified from Sri Lanka aligned with the *S. stercoralis* species according to the available sequences in the NCBI from Australia, Iran and Vietnam (Fig. 3). Therefore, the results confirm that the DNA sequences were homologous to *S. stercoralis*. The new gene sequences were deposited in the NCBI database under the accession numbers OQ923627- OQ923634.

### Serological detection of IgG antibodies

**Fig. 4:**
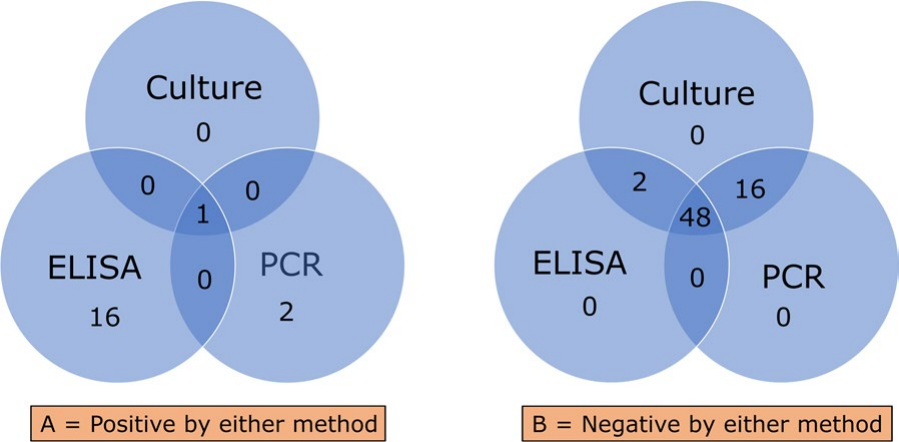
Venn diagram demonstrating the positives (4A) and negatives (4B) by all three tests

Out of the 122 ELISA tests performed among patients with malignancies, 20 patients became positive for *Strongyloides*-specific IgG antibodies. The proportion of seropositivity was 16.4%. Overall, 67 patients underwent all types of diagnostic tests, and the positives and negatives are shown in a Venn diagram in Fig. 4. Only one patient was positive by all three diagnostic methods. Forty-eight patients were negative by all three methods. Sixteen ELISA positives were PCR and culture negative. Two of the PCR positives were ELISA negative.

## Discussion

Strongyloidiasis is a neglected tropical disease caused by the parasitic nematode *Strongyloides stercoralis*. While not extensively studied in Sri Lanka, case reports and a related study has shed light on its presence among specific populations in the country, particularly in immunocompromised individuals [5, 6, 8–10, 15]. Therefore, this study was focussed on detecting the presence of human strongyloidiasis among patients with a high risk of infection-related complications attending selected tertiary care hospitals in Colombo, Sri Lanka.

Our results indicated that 11.4% of our study participants had evidence of DNA of *S. stercoralis* in their faecal samples, whilst 16.4% had evidence of *S. stercoralis*-specific IgG antibodies in their serum. Interestingly, conventional parasitological methods such as direct smear and culture showed low sensitivity in detecting *Strongyloides* infections, with only one sample (0.6%) detected positive by agar plate culture. This highlights the limitations of traditional diagnostic approaches, which has been discussed in the literature [4, 20].

The observed low percentage of strongyloidiasis by copro-parasitological methods may be due to several reasons. Firstly, it is important to consider the changing epidemiology of soil transmitted helminthiases in Sri Lanka. Routine deworming programmes mainly targeting pregnant women and school children, along with improved housing and sanitation has resulted dramatic reduction in the prevalence of STH [21, 22]. On the other hand, the reduced sensitivity of the tests themselves [23] could play a role in the presumed low copro-prevalence. Several studies have illustrated up to two-fold increase in the yield of larvae following agar plate cultures of multiple samples [23]. Therefore, repeated faecal samples are required to increase the sensitivity. However, despite detailed counselling to every single patient recruited to this study and tests being free of charge, the response rate was very low, which has been identified in other similar studies as well [15, 24–26].

Comparisons with existing literature on strongyloidiasis prevalence are essential for contextualizing the findings of this study. While there is limited data specifically focusing on strongyloidiasis prevalence among high-risk patients attending tertiary care hospitals in Colombo, studies from other regions have reported varying prevalence rates. For example, studies conducted in tropical and subtropical regions have reported prevalence rates ranging from 10% to 40% [11]. These variations highlight the complex epidemiology of strongyloidiasis and the influence of geographical, environmental, and demographic factors on disease prevalence.

When considering the subset of patients on whom all three types of diagnostic tests were carried out, 16 patients who were PCR negative were seropositive. This PCR negativity may be due to the reduced larval excretion in chronic strongyloidiasis [27] or the presence of inhibitors within the faecal sample that may inhibit the amplification [28]. Unfortunately, it was not plausible to employ an internal control due to the high cost. Moreover, the DNA extraction kit used in our study was specific for faecal samples, which had an inhibitor removal step. Also, we employed a prolonged incubation with the lysis buffer (refer to Methods); thereby, the presence of inhibition is less likely. In contrast, two PCR positives, in the presence of seronegativity, demonstrates the importance of molecular detection in the presence of immunosuppression. This reiterates the importance of using a combination of investigations for the diagnosis of strongyloidiasis [20, 29].

A seemingly high seroprevalence among the patients with malignancies was observed. However, serology is prone to cross reactions, especially with ascariasis, toxocariasis and filariasis [30]. Moreover, serological tests cannot distinguish between past and present infections [31]. Additionally, in non-endemic settings, a higher number of equivocal ELISA were seen in the presence of haematological/immunological disorders [32]. As the number of serum samples in our study was limited to 122 due to difficulties in procuring test kits, and that too consisted mainly of patients with haematological malignancies, whether the disease status itself gave rise to a positive ELISA result in place of negative parasitological/molecular result cannot be excluded. Therefore, the extent to which serology overestimates the country’s disease burden, when the copro-parasitological techniques underestimate it, remains questionable.

In this study, significant associations were not found with the clinico-demographic factors, exposure risk factors or clinical features. This could imply that classical clinical features such as diarrhoea and eosinophilia may not always be present in immunosuppressed patients with strongyloidiasis, and implies that screening is indicated regardless of the presence of symptoms. On the other hand, the lack of association could possibly be due to the low sample size involved, and has been observed in similar studies with similar sample sizes [15, 18, 33]. Therefore, the study sample size might have limited the ability to detect significant associations. More patients with haematological malignancies were recruited in this study as they were found to be at higher risk of developing strongyloidiasis [34]; however, we could not demonstrate an association with underlying immunosuppressing condition and disease positivity. Despite the lack of association in this study and not being investigated as a separate risk factor for severe strongyloidiasis, infections represent the second commonest cause of death in end stage renal disease [35]. This is particularly important as septic shock is a complication that can occur with severe strongyloidiasis due to massive larval penetration into the intestinal mucosa, accompanied by bacterial translocation [33]. Chronic kidney disease is a burden in Sri Lanka, therefore with increased number of patients progressing towards end stage renal disease and receiving haemodialysis, screening for strongyloidiasis by highly sensitive methods such as PCR, and thereby provision of timely treatment would be beneficial, and thus requires further investigation.

In this study, the sensitivity, specificity, negative predictive value and positive predictive value could not be calculated as only 67 patients underwent all copro-parasitological, serological and molecular investigations. On the other hand, we were not able to collect both faecal and blood samples from all patients recruited for the study. Therefore, it is difficult to make a comparison of techniques used in the present study. Moreover, patients representing the entire country, especially the hill country that is known for having high prevalence of soil-transmitted helminthiases due to poor sanitary conditions, were not sampled in the study. Also, there is no current understanding about the seroprevalence in the immunocompetent population in Sri Lanka. The lack of a control sample could be highlighted as another limitation of this study. Further, all samples were not tested by PCR, ELISA and sequencing due to limitation in funds. However, despite the limitations, the present study highlights the occurrence of strongyloidiasis among immunocompromised individuals in Sri Lanka, emphasizing the importance of using highly sensitive diagnostic methods such as PCR and ELISA for early detection and treatment. Further research involving larger sample sizes and longitudinal studies are warranted to better understand the epidemiology and risk factors associated with strongyloidiasis in Sri Lanka.

## Conclusions

Despite the pandemic and economic challenges, a study in Sri Lanka revealed varying strongyloidiasis infection rates among immunocompromised patients. The percentage of *Strongyloides* specific antibodies by ELISA was 16.4%, likely overestimated due to false positives; copro-parasitological methods indicated 0.6%, potentially underestimated due to poor sensitivity. The presence of *S. stercoralis* DNA in 11.4% is considered the most accurate estimate. The absence of typical clinical features suggests chronic disease, and the lack of associated factors hints at limited parasite exposure. The study underscores the importance of screening immunocompromised patients for strongyloidiasis using molecular methods like PCR to prevent severe, life-threatening consequences.

## Supplementary Information

Additional file 1: Figure S1. Morphology of a *Strongyloides stercoralis* rhabditiform larva extracted from the agar plate culture (×400 magnification) indicating short buccal canal (blue arrow), prominent oesophageal bulb (purple arrow), and prominent genital primordium (green arrow)

Additional file 2: Figure S2. Gel photograph of qualitative PCR positive for the DNA extracted from patient stool samples. Lanes 1–9: patient samples, lane 10: positive control, lane 11: negative control (PCR water), and lane 12: 50 BP ladder

## Data Availability

The data supporting the conclusions of this article are included within the article. The newly generated sequences are were submitted to the GenBank database under the accession numbers OQ923627 – OQ923634.

## Abbreviations

DNA: Deoxyribonucleic acid
ELISA: Enzyme-linked immunosorbent assay
*ITS1*: Internal transcribed spacer 1
PCR: Polymerase chain reaction
RNA: Ribonucleic acid
STH: Soil transmitted helminths/ helminthiases
WHO: World Health Organization
