# Supplementary figures and images for: Detection of human strongyloidiasis among patients with a high risk of complications attending selected tertiary care hospitals in Colombo, Sri Lanka using molecular and serological diagnostic tools
Source: Parasit Vectors. 2024 Oct 12;17:427. doi: 10.1186/s13071-024-06508-x (PMC11470637; doi:10.1186/s13071-024-06508-x)

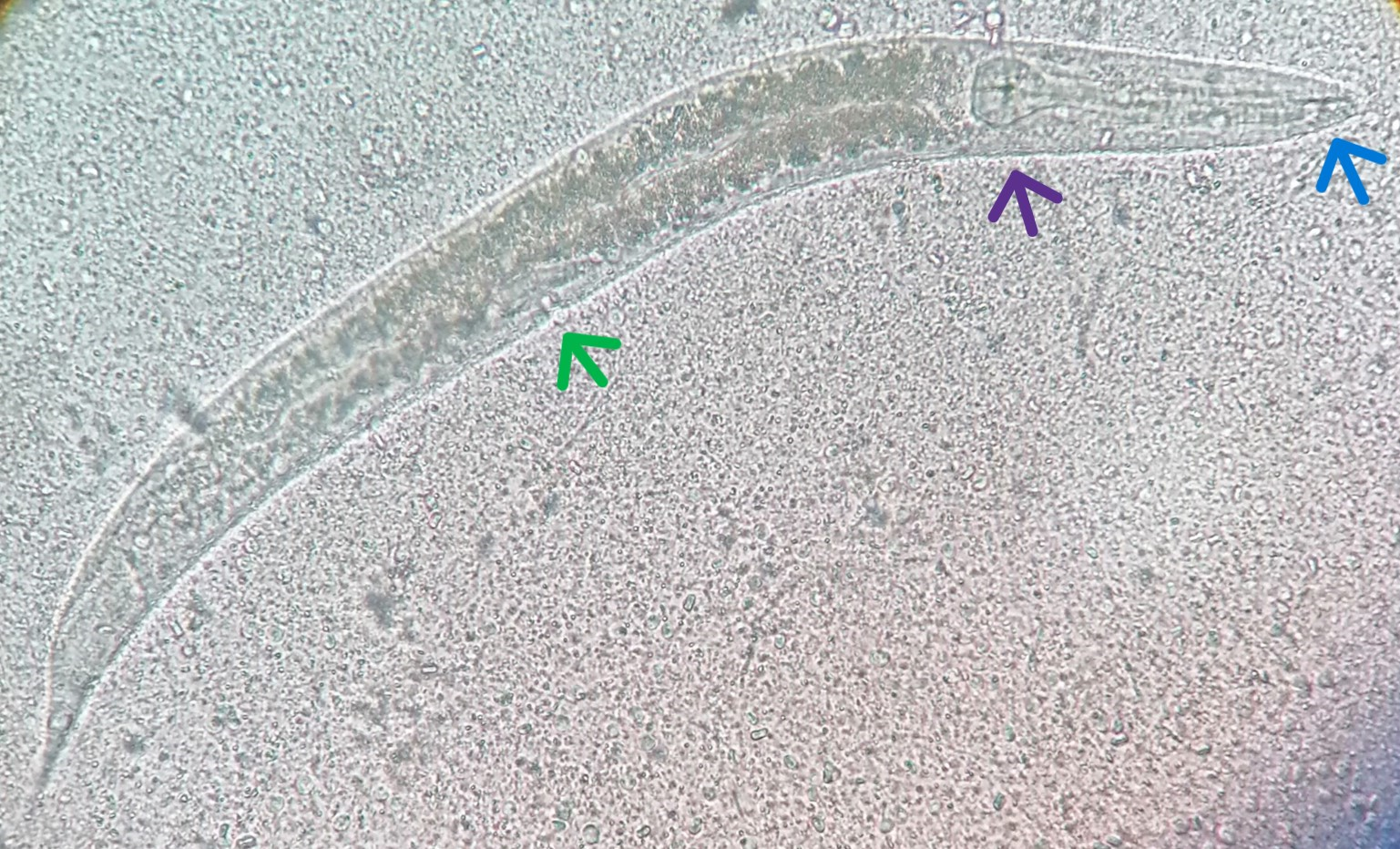

Supplement: Supplementary file 1 — Additional file 1: Figure S1. Morphology of a Strongyloides stercoralis rhabditiform larva extracted from the agar plate culture (×400 magnification) indicating short buccal canal (blue arrow), prominent oesophageal bulb (purple arrow), and prominent genital primordium (green arrow) [file 13071_2024_6508_MOESM1_ESM.tiff]

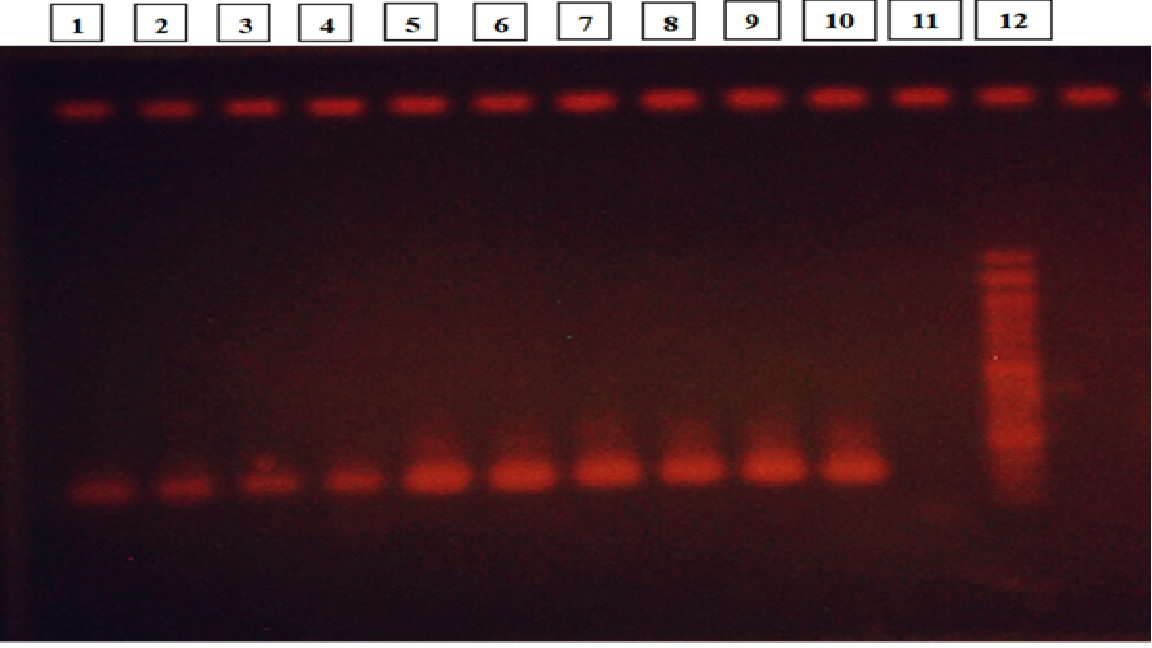

Supplement: Supplementary file 2 — Additional file 2: Figure S2. Gel photograph of qualitative PCR positive for the DNA extracted from patient stool samples. Lanes 1–9: patient samples, lane 10: positive control, lane 11: negative control (PCR water), and lane 12: 50 BP ladder [file 13071_2024_6508_MOESM2_ESM.tiff]
